# Supplementary material for: A Likelihood Approach for Real-Time Calibration of Stochastic Compartmental Epidemic Models
Source: PLoS Comput Biol. 2017 Jan 17;13(1):e1005257. doi: 10.1371/journal.pcbi.1005257 (PMC5240920; doi:10.1371/journal.pcbi.1005257)
Supplement: S1 File — (TAR.GZ) [file pcbi.1005257.s014.tar.gz › HSPH_Online-SI-Revision/MSS10/n1-mild/n1-mild_NEW2_table0.pdf]

1	newly 3 weeks spec	14.	37.23	{21., 52.}	165.929	0.399839	newly 3 weeks cumul	75.	113.6	{74., 150.}	52.96	0.173138	newly 5 weeks spec	14.	31.07	{19., 44.}	122.786	0.328279	newly 5 weeks cumul	112.	179.48	{124., 231.}	60.9107	0.195289	newly 55 weeks cumul	214.	321.82	{248., 407.}	51.7196	0.174312
2	newly 3 weeks spec	8.	8.11	{0., 20.}	84.375	0.704273	newly 3 weeks cumul	39.	48.63	{12., 101.}	64.5385	0.276798	newly 5 weeks spec	6.	2.21	{0., 9.}	89.8333	1.26474	newly 5 weeks cumul	52.	54.82	{12., 110.}	56.8077	0.292916	newly 51 weeks cumul	74.	58.33	{12., 112.}	50.5811	0.338483
3	newly 3 weeks spec	19.	20.68	{0., 52.}	92.7368	0.848097	newly 3 weeks cumul	66.	96.05	{14., 195.}	86.2576	0.348241	newly 5 weeks spec	13.	7.2	{0., 24.}	91.8462	1.29536	newly 5 weeks cumul	88.	115.07	{14., 243.}	77.8295	0.357994	newly 60 weeks cumul	152.	124.84	{14., 280.}	55.1579	0.39204
4	newly 3 weeks spec	7.	19.01	{0., 47.}	230.143	0.833993	newly 3 weeks cumul	27.	77.62	{10., 156.}	212.741	0.497111	newly 5 weeks spec	12.	7.63	{0., 24.}	97.0833	1.31153	newly 5 weeks cumul	48.	97.49	{10., 192.}	143.063	0.462337	newly 54 weeks cumul	336.	114.72	{10., 259.}	66.119	0.712454
5	newly 3 weeks spec	21.	22.33	{0., 47.}	65.6667	0.489527	newly 3 weeks cumul	87.	123.44	{38., 186.}	62.4598	0.227461	newly 5 weeks spec	13.	8.27	{0., 21.}	78.2308	0.979473	newly 5 weeks cumul	118.	146.49	{50., 237.}	51.839	0.214798	newly 56 weeks cumul	175.	154.65	{50., 256.}	36.8743	0.214607
6	newly 3 weeks spec	25.	66.26	{37., 89.}	170.32	0.444197	newly 3 weeks cumul	80.	175.96	{121., 233.}	124.225	0.345955	newly 5 weeks spec	28.	63.58	{20., 90.}	139.643	0.410515	newly 5 weeks cumul	143.	305.92	{199., 391.}	118.769	0.340668	newly 60 weeks cumul	316.	586.73	{275., 763.}	93.6614	0.291431
7	newly 3 weeks spec	43.	34.11	{0., 74.}	57.0465	0.584468	newly 3 weeks cumul	128.	220.42	{29., 365.}	101.078	0.41769	newly 5 weeks spec	19.	5.69	{0., 17.}	86.2632	1.5263	newly 5 weeks cumul	180.	239.01	{29., 376.}	64.1722	0.355915	newly 59 weeks cumul	287.	243.61	{29., 380.}	32.8955	0.308439
8	newly 3 weeks spec	17.	20.83	{5., 40.}	63.9412	0.272565	newly 3 weeks cumul	45.	59.82	{21., 114.}	65.5111	0.241109	newly 5 weeks spec	19.	18.66	{4., 36.}	55.7895	0.337159	newly 5 weeks cumul	80.	98.99	{38., 177.}	59.1125	0.230989	newly 51 weeks cumul	385.	275.67	{100., 404.}	31.6597	0.220406
9	newly 3 weeks spec	39.	19.35	{0., 39.}	55.7179	0.636428	newly 3 weeks cumul	109.	82.13	{19., 141.}	41.5872	0.296214	newly 5 weeks spec	28.	8.13	{0., 21.}	76.4643	1.25491	newly 5 weeks cumul	160.	103.11	{19., 177.}	43.3563	0.343789	newly 50 weeks cumul	206.	116.99	{19., 207.}	48.9951	0.406125
10	newly 3 weeks spec	14.	33.02	{23., 44.}	136.571	0.362078	newly 3 weeks cumul	60.	107.24	{84., 123.}	78.9	0.247174	newly 5 weeks spec	11.	25.79	{17., 35.}	134.455	0.354278	newly 5 weeks cumul	94.	161.51	{128., 187.}	72.0957	0.230891	newly 51 weeks cumul	109.	293.7	{253., 346.}	169.45	0.421957
11	newly 3 weeks spec	16.	29.45	{10., 53.}	100.313	0.311895	newly 3 weeks cumul	57.	85.97	{38., 157.}	67.2456	0.228656	newly 5 weeks spec	6.	23.58	{6., 47.}	307.333	0.627919	newly 5 weeks cumul	78.	135.98	{55., 255.}	89.0769	0.28053	newly 50 weeks cumul	387.	254.39	{60., 384.}	36.3902	0.28733
12	newly 3 weeks spec	18.	26.5	{0., 58.}	108.333	0.720495	newly 3 weeks cumul	54.	89.78	{11., 157.}	102.185	0.384581	newly 5 weeks spec	20.	14.47	{0., 46.}	90.15	1.14172	newly 5 weeks cumul	85.	125.9	{11., 246.}	90.7294	0.397495	newly 51 weeks cumul	271.	167.62	{11., 374.}	56.5018	0.518018
13	newly 3 weeks spec	19.	29.04	{21., 37.}	55.0526	0.185151	newly 3 weeks cumul	55.	97.1	{82., 110.}	76.5455	0.244299	newly 5 weeks spec	8.	23.21	{15., 31.}	193.625	0.472949	newly 5 weeks cumul	79.	146.93	{121., 167.}	85.9873	0.26606	newly 52 weeks cumul	97.	279.63	{236., 333.}	188.485	0.453177
14	newly 3 weeks spec	15.	22.43	{3., 41.}	84.2	0.422809	newly 3 weeks cumul	78.	84.77	{34., 142.}	43.7821	0.208631	newly 5 weeks spec	10.	12.03	{0., 29.}	87.3	0.620252	newly 5 weeks cumul	112.	114.14	{36., 190.}	42.1071	0.223258	newly 56 weeks cumul	131.	138.02	{40., 227.}	46.1069	0.243
15	newly 3 weeks spec	27.	19.95	{1., 39.}	53.2963	0.509023	newly 3 weeks cumul	78.	86.2	{26., 156.}	52.4615	0.256892	newly 5 weeks spec	19.	8.96	{0., 27.}	69.7895	1.02415	newly 5 weeks cumul	124.	109.62	{27., 199.}	44.5161	0.281126	newly 56 weeks cumul	280.	121.91	{27., 232.}	56.7893	0.48979
16	newly 3 weeks spec	11.	22.01	{13., 33.}	102.818	0.288278	newly 3 weeks cumul	35.	65.45	{47., 83.}	87.	0.2581	newly 5 weeks spec	16.	22.16	{13., 31.}	46.	0.155922	newly 5 weeks cumul	59.	110.51	{80., 145.}	87.3051	0.259973	newly 54 weeks cumul	273.	378.49	{291., 459.}	39.4469	0.139183
17	newly 3 weeks spec	20.	29.99	{15., 46.}	60.15	0.197463	newly 3 weeks cumul	50.	82.68	{54., 119.}	68.24	0.212466	newly 5 weeks spec	12.	34.45	{15., 57.}	189.917	0.423907	newly 5 weeks cumul	78.	150.62	{91., 215.}	94.9744	0.271759	newly 57 weeks cumul	269.	453.14	{179., 666.}	80.9591	0.255351
18	newly 3 weeks spec	14.	21.76	{7., 37.}	74.7143	0.264476	newly 3 weeks cumul	53.	66.98	{30., 107.}	45.3208	0.17404	newly 5 weeks spec	18.	17.9	{4., 33.}	50.	0.322276	newly 5 weeks cumul	81.	105.26	{44., 163.}	47.4568	0.182556	newly 50 weeks cumul	239.	192.95	{49., 308.}	34.046	0.224778
19	newly 3 weeks spec	24.	21.15	{4., 35.}	45.5417	0.34769	newly 3 weeks cumul	86.	89.32	{29., 147.}	40.814	0.217532	newly 5 weeks spec	24.	7.84	{0., 17.}	69.5	0.961661	newly 5 weeks cumul	128.	110.53	{38., 174.}	36.1953	0.229	newly 57 weeks cumul	218.	125.31	{43., 200.}	44.5	0.33856
20	newly 3 weeks spec	15.	39.38	{22., 55.}	168.267	0.431685	newly 3 weeks cumul	66.	124.47	{74., 148.}	93.1667	0.281296	newly 5 weeks spec	18.	31.29	{12., 46.}	89.7222	0.349957	newly 5 weeks cumul	102.	190.89	{117., 234.}	93.5784	0.289857	newly 54 weeks cumul	153.	374.46	{132., 489.}	154.405	0.406101
21	newly 3 weeks spec	19.	14.11	{0., 32.}	66.3684	0.774967	newly 3 weeks cumul	59.	83.19	{20., 157.}	74.7627	0.303559	newly 5 weeks spec	16.	4.1	{0., 13.}	82.875	1.49459	newly 5 weeks cumul	93.	94.94	{20., 179.}	54.6452	0.308691	newly 54 weeks cumul	145.	100.33	{20., 190.}	47.3586	0.365809
22	newly 3 weeks spec	17.	25.37	{15., 38.}	56.7647	0.182625	newly 3 weeks cumul	45.	67.19	{42., 91.}	54.2889	0.176458	newly 5 weeks spec	12.	30.18	{15., 45.}	153.5	0.369572	newly 5 weeks cumul	68.	124.37	{78., 163.}	85.3676	0.249474	newly 56 weeks cumul	388.	555.97	{400., 664.}	48.0232	0.170971
23	newly 3 weeks spec	18.	18.29	{1., 37.}	66.3889	0.47691	newly 3 weeks cumul	69.	74.58	{22., 128.}	48.6667	0.231691	newly 5 weeks spec	27.	9.11	{0., 23.}	71.1481	1.12169	newly 5 weeks cumul	118.	95.84	{23., 155.}	40.9153	0.260045	newly 51 weeks cumul	241.	118.4	{23., 207.}	52.5228	0.428711
24	newly 3 weeks spec	25.	39.52	{29., 49.}	59.36	0.195016	newly 3 weeks cumul	83.	113.7	{91., 132.}	37.5181	0.135018	newly 5 weeks spec	20.	40.04	{28., 51.}	101.	0.293125	newly 5 weeks cumul	121.	194.86	{162., 227.}	61.5207	0.205156	newly 53 weeks cumul	298.	537.45	{489., 589.}	80.3523	0.254769
25	newly 3 weeks spec	12.	19.25	{7., 32.}	79.75	0.273744	newly 3 weeks cumul	34.	57.08	{26., 94.}	78.1765	0.235513	newly 5 weeks spec	11.	17.38	{4., 32.}	94.3636	0.374535	newly 5 weeks cumul	59.	92.07	{40., 151.}	71.2712	0.229396	newly 50 weeks cumul	239.	248.49	{81., 450.}	47.251	0.238566
26	newly 3 weeks spec	22.	32.14	{21., 43.}	51.1818	0.172625	newly 3 weeks cumul	61.	98.52	{79., 118.}	62.1311	0.205019	newly 5 weeks spec	13.	26.55	{14., 35.}	109.923	0.323283	newly 5 weeks cumul	91.	154.78	{123., 187.}	71.2747	0.228624	newly 53 weeks cumul	122.	327.33	{225., 435.}	169.861	0.421832
27	newly 3 weeks spec	11.	34.52	{23., 44.}	213.818	0.482698	newly 3 weeks cumul	44.	95.5	{75., 114.}	117.045	0.330615	newly 5 weeks spec	10.	37.43	{25., 50.}	274.3	0.556899	newly 5 weeks cumul	72.	169.87	{137., 202.}	135.931	0.367038	newly 50 weeks cumul	169.	499.52	{447., 555.}	196.296	0.46832
28	newly 3 weeks spec	15.	38.72	{26., 50.}	158.133	0.396232	newly 3 weeks cumul	48.	101.19	{79., 124.}	110.813	0.317116	newly 5 weeks spec	16.	46.88	{36., 59.}	193.	0.458667	newly 5 weeks cumul	77.	192.14	{156., 228.}	149.532	0.391805	newly 56 weeks cumul	293.	680.4	{629., 731.}	132.218	0.365059
29	newly 3 weeks spec	19.	35.9	{17., 49.}	100.211	0.333732	newly 3 weeks cumul	60.	115.59	{61., 144.}	100.383	0.313333	newly 5 weeks spec	14.	28.81	{6., 43.}	126.929	0.441711	newly 5 weeks cumul	96.	176.53	{95., 220.}	93.5104	0.306385	newly 53 weeks cumul	143.	351.54	{129., 473.}	157.245	0.421801
30	newly 3 weeks spec	28.	31.4	{13., 50.}	42.9286	0.209036	newly 3 weeks cumul	82.	105.42	{59., 151.}	42.0244	0.16544	newly 5 weeks spec	11.	21.55	{5., 38.}	116.091	0.382673	newly 5 weeks cumul	114.	153.67	{81., 228.}	48.1316	0.187319	newly 57 weeks cumul	214.	233.34	{118., 329.}	33.4206	0.168242
31	newly 3 weeks spec	34.	51.83	{42., 63.}	52.9118	0.17957	newly 3 weeks cumul	96.	170.12	{152., 189.}	77.2083	0.246439	newly 5 weeks spec	13.	38.5	{30., 47.}	196.154	0.463868	newly 5 weeks cumul	127.	254.12	{228., 280.}	100.094	0.299369	newly 55 weeks cumul	143.	421.67	{377., 468.}	194.874	0.467164
32	newly 3 weeks spec	11.	29.41	{12., 45.}	173.	0.405219	newly 3 weeks cumul	53.	84.79	{39., 115.}	71.1509	0.229954	newly 5 weeks spec	12.	29.83	{12., 44.}	154.583	0.3835	newly 5 weeks cumul	70.	145.14	{68., 201.}	111.4	0.304532	newly 53 weeks cumul	339.	420.84	{244., 538.}	40.1711	0.164444
33	newly 3 weeks spec	38.	35.59	{21., 50.}	24.7632	0.117983	newly 3 weeks cumul	103.	112.29	{76., 149.}	20.5922	0.0824199	newly 5 weeks spec	18.	28.85	{16., 42.}	67.1667	0.207402	newly 5 weeks cumul	138.	173.88	{125., 225.}	31.2754	0.113167	newly 54 weeks cumul	205.	299.03	{202., 375.}	50.1805	0.166213
34	newly 3 weeks spec	13.	17.69	{0., 44.}	107.615	0.706492	newly 3 weeks cumul	28.	60.81	{10., 121.}	150.536	0.404367	newly 5 weeks spec	11.	10.26	{0., 29.}	99.6364	1.06015	newly 5 weeks cumul	49.	84.36	{10., 188.}	118.694	0.416466	newly 53 weeks cumul	176.	114.49	{10., 251.}	57.858	0.522154
35	newly 3 weeks spec	36.	50.21	{40., 62.}	42.4167	0.149618	newly 3 weeks cumul	89.	146.35	{121., 166.}	64.7079	0.211702	newly 5 weeks spec	34.	44.83	{31., 57.}	35.7353	0.129088	newly 5 weeks cumul	162.	239.99	{201., 270.}	48.9444	0.169569	newly 55 weeks cumul	281.	534.48	{409., 591.}	90.3488	0.274923
36	newly 3 weeks spec	29.	55.76	{41., 70.}	93.0345	0.278052	newly 3 weeks cumul	101.	172.94	{151., 194.}	71.2277	0.230317	newly 5 weeks spec	18.	47.72	{37., 59.}	167.111	0.437884	newly 5 weeks cumul	133.	273.11	{240., 307.}	105.346	0.309237	newly 57 weeks cumul	143.	494.03	{453., 533.}	245.476	0.534194
37	newly 3 weeks spec	16.	25.82	{4., 47.}	92.	0.40485	newly 3 weeks cumul	67.	96.13	{36., 167.}	68.8209	0.248925	newly 5 weeks spec	19.	12.82	{0., 29.}	62.2105	0.670479	newly 5 weeks cumul	103.	128.49	{39., 221.}	58.3398	0.254376	newly 53 weeks cumul	179.	167.77	{39., 275.}	42.352	0.265796
38	newly 3 weeks spec	31.	34.38	{0., 65.}	70.6452	0.555297	newly 3 weeks cumul	127.	135.53	{40., 201.}	43.2205	0.224152	newly 5 weeks spec	22.	21.83	{0., 54.}	95.8636	1.03096	newly 5 weeks cumul	168.	186.93	{48., 312.}	54.5893	0.275237	newly 57 weeks cumul	211.	275.72	{48., 566.}	83.1943	0.361285
39	newly 3 weeks spec	16.	18.86	{4., 35.}	60.	0.331822	newly 3 weeks cumul	52.	77.15	{28., 127.}	68.75	0.251518	newly 5 weeks spec	14.	9.55	{0., 21.}	57.0714	0.546651	newly 5 weeks cumul	79.	100.12	{34., 164.}	51.8481	0.222247	newly 55 weeks cumul	143.	119.08	{36., 207.}	37.0629	0.236695
40	newly 3 weeks spec	28.	24.44	{6., 47.}	50.9286	0.366717	newly 3 weeks cumul	115.	116.51	{41., 190.}	41.2783	0.214775	newly 5 weeks spec	12.	8.55	{0., 25.}	78.0833	0.881517	newly 5 weeks cumul	156.	140.8	{55., 224.}	38.5256	0.222801	newly 58 weeks cumul	187.	160.	{56., 275.}	41.0374	0.248051
41	newly 3 weeks spec	19.	28.26	{0., 56.}	105.684	0.644648	newly 3 weeks cumul	73.	116.9	{25., 196.}	89.4521	0.321389	newly 5 weeks spec	10.	14.46	{0., 43.}	150.	1.14071	newly 5 weeks cumul	107.	150.44	{25., 262.}	79.1589	0.334669	newly 59 weeks cumul	145.	195.25	{25., 434.}	83.8	0.370584
42	newly 3 weeks spec	12.	49.44	{17., 74.}	318.	0.615123	newly 3 weeks cumul	104.	163.35	{74., 213.}	68.8558	0.241979	newly 5 weeks spec	0.1	38.33	{3., 67.}	38236.	2.38058	newly 5 weeks cumul	106.	244.07	{121., 328.}	137.047	0.369607	newly 58 weeks cumul	106.	400.45	{135., 633.}	284.123	0.537838
43	newly 3 weeks spec	28.	22.42	{4., 42.}	44.7143	0.384338	newly 3 weeks cumul	117.	116.13	{56., 181.}	32.3675	0.164274	newly 5 weeks spec	11.	7.02	{0., 17.}	70.	0.789142	newly 5 weeks cumul	151.	135.36	{63., 209.}	30.7152	0.170472	newly 60 weeks cumul	167.	142.45	{71., 219.}	30.8204	0.178514
44	newly 3 weeks spec	20.	20.32	{0., 40.}	68.1	0.54809	newly 3 weeks cumul	78.	96.62	{31., 165.}	58.8205	0.275743	newly 5 weeks spec	6.	8.06	{0., 23.}	132.	0.96562	newly 5 weeks cumul	92.	117.27	{31., 214.}	64.3152	0.300169	newly 52 weeks cumul	103.	126.98	{31., 233.}	64.1359	0.311143
45	newly 3 weeks spec	25.	18.96	{1., 41.}	54.64	0.502333	newly 3 weeks cumul	80.	74.34	{12., 129.}	45.5	0.267781	newly 5 weeks spec	16.	9.66	{0., 25.}	61.125	0.779803	newly 5 weeks cumul	114.	97.73	{12., 181.}	43.2544	0.286238	newly 50 weeks cumul	184.	120.51	{12., 218.}	46.7772	0.368704
46	newly 3 weeks spec	21.	41.75	{29., 58.}	102.143	0.311331	newly 3 weeks cumul	65.	112.36	{77., 148.}	75.5385	0.237428	newly 5 weeks spec	19.	40.98	{22., 57.}	120.421	0.363219	newly 5 weeks cumul	90.	195.37	{130., 251.}	119.922	0.336806	newly 57 weeks cumul	370.	513.44	{326., 622.}	48.973	0.191376
47	newly 3 weeks spec	10.	31.14	{20., 42.}	211.4	0.476492	newly 3 weeks cumul	30.	78.19	{58., 97.}	160.633	0.406922	newly 5 weeks spec	5.	38.35	{26., 51.}	667.	0.869102	newly 5 weeks cumul	45.	150.62	{108., 189.}	234.711	0.515518	newly 55 weeks cumul	300.	656.52	{594., 709.}	118.84	0.339102
48	newly 3 weeks spec	18.	29.11	{22., 36.}	62.5	0.202757	newly 3 weeks cumul	78.	89.21	{76., 102.}	15.5769	0.0609124	newly 5 weeks spec	4.	26.54	{17., 36.}	563.5	0.806709	newly 5 weeks cumul	93.	143.44	{122., 163.}	54.2366	0.185536	newly 50 weeks cumul	172.	387.23	{343., 425.}	125.134	0.350649
49	newly 3 weeks spec	6.	26.97	{12., 40.}	350.5	0.617612	newly 3 weeks cumul	35.	72.77	{44., 99.}	109.686	0.306593	newly 5 weeks spec	12.	31.33	{15., 48.}	166.75	0.405511	newly 5 weeks cumul	58.	133.61	{79., 184.}	132.431	0.351518	newly 54 weeks cumul	236.	433.2	{338., 513.}	85.2119	0.264305
50	newly 3 weeks spec	20.	25.94	{15., 36.}	40.6	0.170353	newly 3 weeks cumul	56.	79.45	{64., 104.}	45.5536	0.170385	newly 5 weeks spec	23.	22.53	{12., 33.}	29.6087	0.176898	newly 5 weeks cumul	95.	125.64	{91., 161.}	37.3053	0.150453	newly 51 weeks cumul	220.	331.89	{147., 456.}	64.3318	0.237968
